# A Novel Algorithm for Movement Artifact Removal in ECG Signals Acquired from Wearable Systems Applied to Horses

**DOI:** 10.1371/journal.pone.0140783

**Published:** 2015-10-20

**Authors:** Antonio Lanata, Andrea Guidi, Paolo Baragli, Gaetano Valenza, Enzo Pasquale Scilingo

**Affiliations:** 1 Research Center E. Piaggio, School of Engineering, University of Pisa, Pisa, Italy; 2 Department of Veterinary Sciences, University of Pisa, Pisa, Italy; Aristotle University of Thessaloniki, GREECE

## Abstract

This study reports on a novel method to detect and reduce the contribution of movement artifact (MA) in electrocardiogram (ECG) recordings gathered from horses in free movement conditions. We propose a model that integrates cardiovascular and movement information to estimate the MA contribution. Specifically, ECG and physical activity are continuously acquired from seven horses through a wearable system. Such a system employs completely integrated textile electrodes to monitor ECG and is also equipped with a triaxial accelerometer for movement monitoring. In the literature, the most used technique to remove movement artifacts, when noise bandwidth overlaps the primary source bandwidth, is the adaptive filter. In this study we propose a new algorithm, hereinafter called Stationary Wavelet Movement Artifact Reduction (SWMAR), where the Stationary Wavelet Transform (SWT) decomposition algorithm is employed to identify and remove movement artifacts from ECG signals in horses. A comparative analysis with the Normalized Least Mean Square Adaptive Filter technique (NLMSAF) is performed as well. Results achieved on seven hours of recordings showed a reduction greater than 40% of MA percentage (between before- and after- the application of the proposed algorithm). Moreover, the comparative analysis with the NLMSAF, applied to the same ECG recordings, showed a greater reduction of MA percentage in favour of SWMAR with a statistical significant difference (*p*–*value* < 0.0.5).

## Introduction

Detection and reduction of noises and artifacts in the cardiovascular monitoring are two of the greatest challenges for enhancing signal quality [1–6]. ECG morphology is strongly affected by movement artifacts (MAs), which must be therefore effectively removed [7, 8]. In humans, several efforts have been made to obtain robust ECG against MAs [9–14], but MAs remain one of the major problems in both short- and long- term cardiovascular monitoring [15–18]. More specifically, MAs are mostly generated by skin stretching [19] that is recognized to be the most common source of MAs [20]. Skin stretching causes modifications in the distribution of charges at the interface between the skin and electrode. Since skin can be considered a current generator that actively produces a potential difference between the in- and out-side of the skin, stretching causes disturbance in the distribution of charges at the electrode-electrolyte interface. It induces a temporary change in the half-cell potential, providing a variation of the involved potentials and reducing its magnitude [21–23]. There are two possible approaches to compensating MAs. The first, is the modification of supports and materials involved in the skin-electrode interface, while the second implements models and algorithms for reducing MA contribution. The first approach is based on reducing skin-electrode discontinuity. It is achieved by cleaning or grazing the part of the skin in contact with the electrode and modifying material or form of the electrode itself [24–26]. An example of the latter approach is the use of textile electrodes, which are made of conductive yarns integrated into textile substrates. They exhibit the advantage of having a bigger surface in contact with the skin than the standard electrodes decreasing the skin-electrode impedance. Moreover, textile electrodes offer a greater dynamical adaptation to anatomical profiles [24]. The second approach includes procedures and algorithms which aim at detecting and removing artifacts from the acquired signals. Several techniques have been used in biomedical applications compensate for MAs. Among these, one of the most used and effective is the adaptive filtering cancellation technique [27–29]. It uses a “primary” input containing the corrupted signal (signal with artifacts) and a “reference” input containing artifacts correlated in some unknown way with the primary noise [30]. This adaptive filtering adjusts its parameters automatically, requiring a little a priori knowledge of noise characteristics. More specifically, the reference input is derived from a noise field where the signal is weak or undetectable, and is subtracted from a primary input containing both signal and noise. In this way, the primary noise is attenuated or eliminated by cancellation. Successive implementations were then improved with the introduction of recursivity [31]. In this latter case, an adaptive recurrent filter structure was introduced where the primary input was the ECG signal affected by MAs, while the reference input was an impulse train, whose impulses were coincident with the onsets of QRS complexes. This approach has two major limitations. The first is the introduction of errors due to the inaccurate coincidence of the impulses of the reference signal with the QRS complex onsets. The second issue is related to unusual beat-by-beat variation that may prevent complete adaptation from taking place [31]. Further studies evidenced the importance of acquiring noise, movement, as well as skin-electrode impedance. In fact, some studies on the correlation between MAs and electrode impedance have been already done in the literature [32]. Other studies have introduced stretch sensors to measure MAs effect [33, 34]. In such cases, both electrode-skin impedance and sensor were mounted on the electrode for adaptively modeling motion artifact. In some cases, the need to measure the abovementioned parameters led to the development of novel hardware prototypes [1]. Other researchers proposed to measure the voltage difference from two adjacent electrodes to estimate a noise reference signal [35]. Furthermore, skin stretch was also estimated by using non-invasive optical sensors incorporated into ECG electrodes [36]. The reported research findings have underlined the importance of measuring movements for investigating their relationship with the MAs in ECG. In fact, the information obtained from accelerometer and magneto-resistive sensors were recently used [37, 38]. Moreover, other techniques such as principal component (PCA) and independent component (ICA) analysis [39–42] as well as Empirical Mode Decomposition (EMD) [43, 44] have been explored, but nevertheless the MA issue needs to be still addressed. In this work we propose a novel algorithm based on the stationary wavelet transform (SWT) [45] method for robustly estimating MAs for reliable removal. Moreover, it overcomes many limitations due to empirical thresholds proposed by previous research [46].

### The issues of equine ECG traces

The assessment of ECG morphology plays a paramount role in the investigation of cardiac activity in horses [47]. It is important for the evaluation of heart activity during exercise and recovery phase, as well as in the diagnosis of poor performance and exercise intolerance [48–51]. It has been suggested that both sopraventricular and ventricular premature depolarisation during physical activity can be considered a clue of poor performance in the horses [52]. Given the above considerations, high-quality ECG recordings become critical in the field of equine internal and sport medicine and exercise physiology. Therefore, the presence of MAs in the ECG signal should be avoided, since they would make ECG traces useless or misleading. In human cardiology, such issues are solved, or reduced, by limiting the amount of physical activity. Unlike humans, horses never stay still, and MAs are normally present during ECG recording, corrupting a large part of the significant signal [53]. Typically, restraining horse is discouraged because it is unnatural and stressful, and increases the sympathetic contribution to heart control [54], thus giving a misleading ECG interpretation [48]. The problems related to MAs in the equine ECG become more relevant during exercise [55]. To reduce MAs, one of the most adopted strategies is the repositioning of electrodes by modifying the base-apex configuration [47, 56–60]. Despite technical strategies (e.g., glue, adhesive bendage, etc) and the portability achieved by miniaturized holter ECG acquisition platforms, the ECG-related artifacts are even more important in equine sports [47, 53]. Therefore, horse body movements still remain the biggest source of artifacts in ECG, and manual inspection remains the most diffuse method of removing artifacts [57–59], although it is operator-expertise dependent, inaccurate and time-demanding. When manual inspection occurs, the corrupted ECG segments are usually completely removed, thus leading to a great loss of signal and related information [58]. Hence, an optimized approach for movement-related artifact reduction is strongly required in both equine medicine and in the research applied to horses. Unfortunately, there are currently no accurate and consistent algorithms for identifying equine QRS complexes regrading horses.

In this study, we perform an automatic movement artifact identification and removal of corrupted ECGs through innovative algorithms based on the discrete stationary wavelet transform (SWT). Thanks to a wearable monitoring system, ECG signal and physical activity were continuously monitored to be then combined and put through a multi-resolution thresholding procedure to obtain a signal that was and estimate of the MAs. Finally, by using a simple subtracting process, we removed MAs from the original ECGs. Results are compared to those coming from the application of the Normalized Least Mean Square Adaptive Filter method (NLMSAF) on the same ECG recordings. The manuscript is organized as follows: Section II describes the experimental protocol, along with the signal processing chain. The sections relative to the signal processing chain are split into three sub paragraphs: (i) preprocessing phase, (ii) detection phase, and (iii) artifact removal phase; Section III results are reported, and in Section IV conclusions are drawn and discussed.

## Materials and Methods

**Fig 1:**
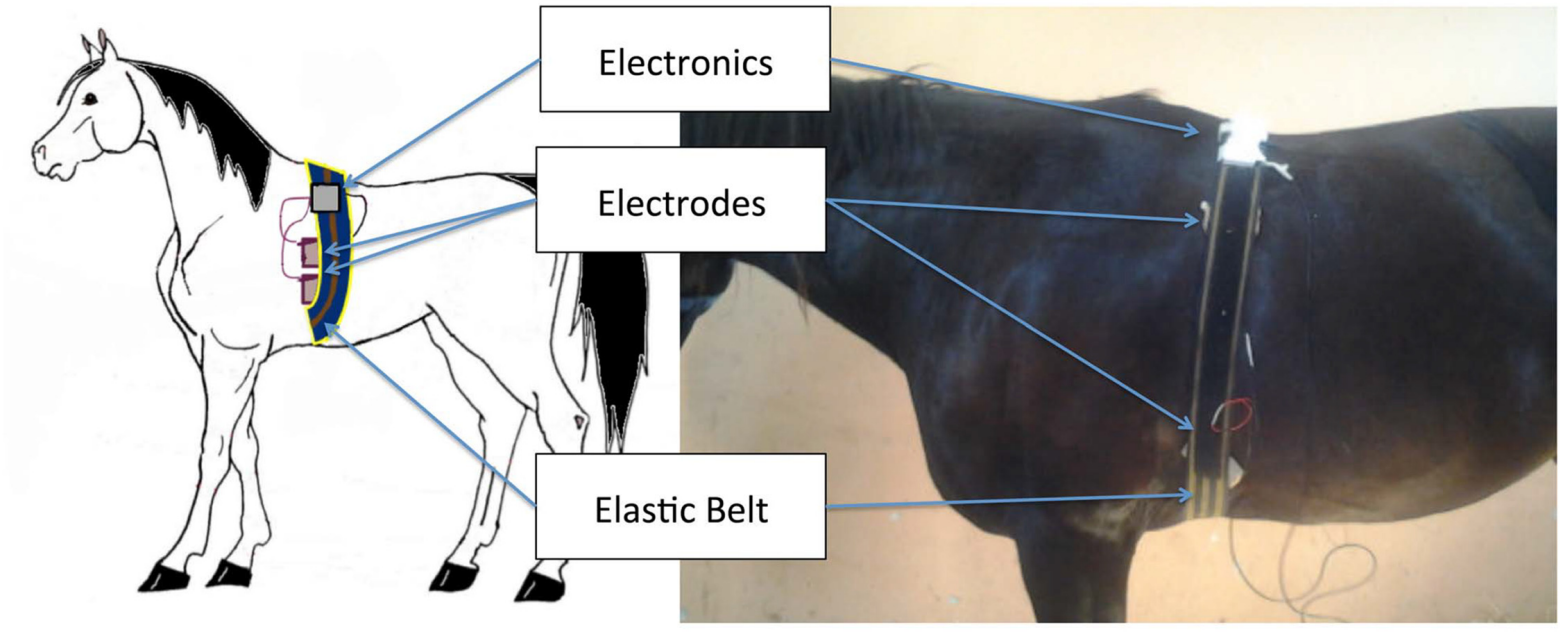
Scheme of the electronic set up: placement of the proposed system.

Seven healthy standardbred mares in anestrus were enrolled in this study (mean age: 8.4 ± 1.3 years, weight: 566.7 ± 54.5 kg. height: 162.9 ± 5.2 cm, body score: 3.9 ± 0.7). Horses were socially housed in a paddock (75 × 75 m) and were provided ad libitum access to both hay and water. Horses were used as receivers in the embryo transfer program of the Department of Veterinary Sciences (University of Pisa, Italy) where this study was performed. Mares were in healthy conditions and not pregnant at the time of this protocol. Each horse underwent an accurate cardiological examination, including ECG and echo-cardio in order to exclude any abnormality in the heart activity. Moreover, they were equipped with a wearable system to monitor physiological signals and physical activity. The wearable monitoring system used in this study (Fig 1) was developed by Smartex s.r.l (Italy) and was comprised of an elastic belt fastened around the chest behind the shoulder area of the horses. Two textile electrodes and a strain gauge sensor were integrated into the belt to acquire the ECG (with a sampling frequency of 250 Hz) in the modified base-apex configuration [56] and respiration activity. The two sensors were finally connected to portable electronics. A triaxial accelerometer was embedded into the electronics, which was positioned on the back of the horse and used to monitor physical activity (with a sampling frequency of 25 Hz). Moreover, through a wireless connection, the system was connected to a mobile device where a dedicated application allowed for checking both the electrode status and the quality of the acquired signal and controlling remotely the storing process in a secure digital (SD) card. Data acquisition was performed in a stall (4 × 4 m), where each horse was alone and left free to move for 60 minutes continuously. The horses were unfed for at least one hour before data acquisition. All horses came from a breeding farm where were used as stud mares after participating in sport. Therefore all horses were perfectly accustomed human handling. At the end of acquisition, and before the application of the processing algorithms, all the acquired ECGs were visually examined by two experts, and all the ECG segments, corrupted by MA, were marked and then used for further validation. The experts were two cardiologists of the Veterinary Teaching Hospital equine clinical section especially trained to individuate MAs. The output of the labeling process was the MA percentage estimated as the percentage of the time in which the ECG exhibits MAs over the whole length of the ECG. Moreover, the experts analyzed the same ECG traces, separately, and in the case of different labelling, an additional veterinary cardiologist was involved to solve the discordant evaluation and to come to a shared and accepted decision in assigning the definitive labels. All raw data can be downloaded as supporting information in S1 Supporting Information.

### Ethics Statement

This study was carried out in accordance with the recommendations in the Italian Animal Care Act (Decree Law 116/92). The experimental protocol was approved by the Ethics Committee on Animal Experimentation of the University of Pisa (reference no. 4886). Consent to participation in the test was signed by each horse owner.

### Signal Processing

**Fig 2:**
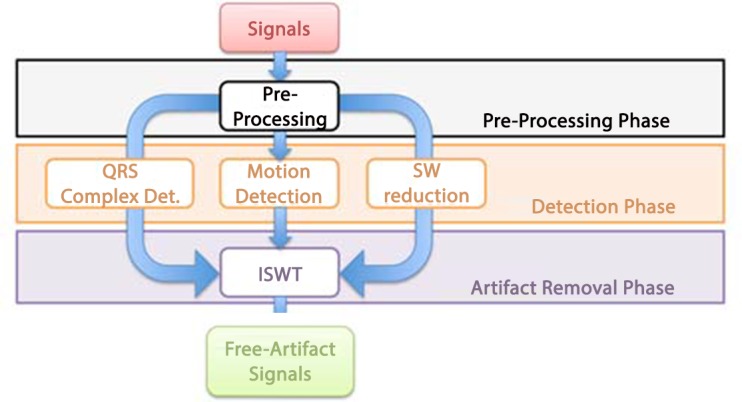
Block diagram of the proposed method. After a pre-processing phase, the SWT decomposition, the QRS complexes and motion detections are performed followed by the reconstruction of the signal affected by artifacts, that is used to reduce and/or remove MAs.

The method here proposed is organised into three different phases. Fig 2 shows a concise scheme of the method. In the first phase, named Preprocessing Phase (PP), all the acquired signals, specifically ECG and accelerometers, were limited in bandwidth. The second phase, named Detection Phase (DP), was comprised of algorithms of QRS complex detection, motion detection, and stationary wavelet reduction. Finally, the third phase, named Artifact Removal Phase (ARP), was focused on the reconstruction of a signal which includes only artifacts, successively removed from the starting raw ECG. In the next, the whole signal processing chain is explained in detail. Hereinafter the whole method is named Stationary Wavelet Movement Artifact Reduction (SWMAR).

#### Preprocessing Phase

In this phase, ECG signals are digitally filtered first with a zero-phase Butterworth IIR band pass filter (0.5–40 Hz), while the accelerometer signals are up-sampled, by means of a linear interpolation method, from 25 Hz to 250 Hz, and combined in order to obtain the acceleration module vectors according to Eq (1)

$$\begin{array}{c} ACC=\sqrt{acc_{x}^{2}+acc_{y}^{2}+acc_{z}^{2}} \end{array}$$ (1)

where *ACC* is the acceleration module vector and *acc*_*x*_, *acc*_*y*_, *acc*_*z*_ are the accelerometer components.

#### Detection Phase

This phase has three steps: QRS complex detection, detection of the ACC segments in which the movement was supposed to introduce artifacts into the ECG signals, and finally the ECG signal decomposition through SWT.

**Fig 3:**
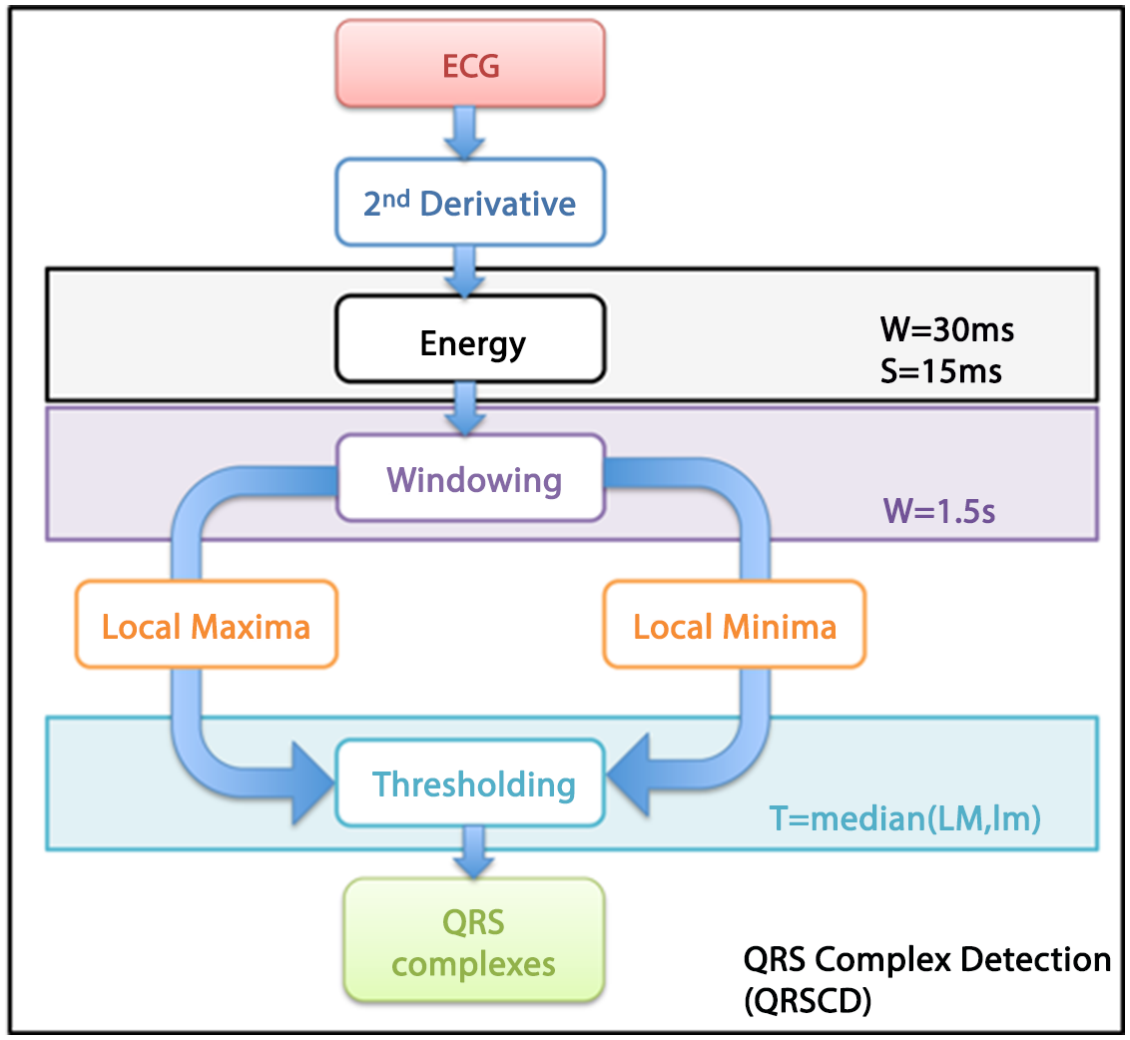
Block diagram of the QRS-complexes detection. The method employes a threshold to detect QRS complexes based on an estimate of the energy of the second derivative of the ECG signals.

**Fig 4:**
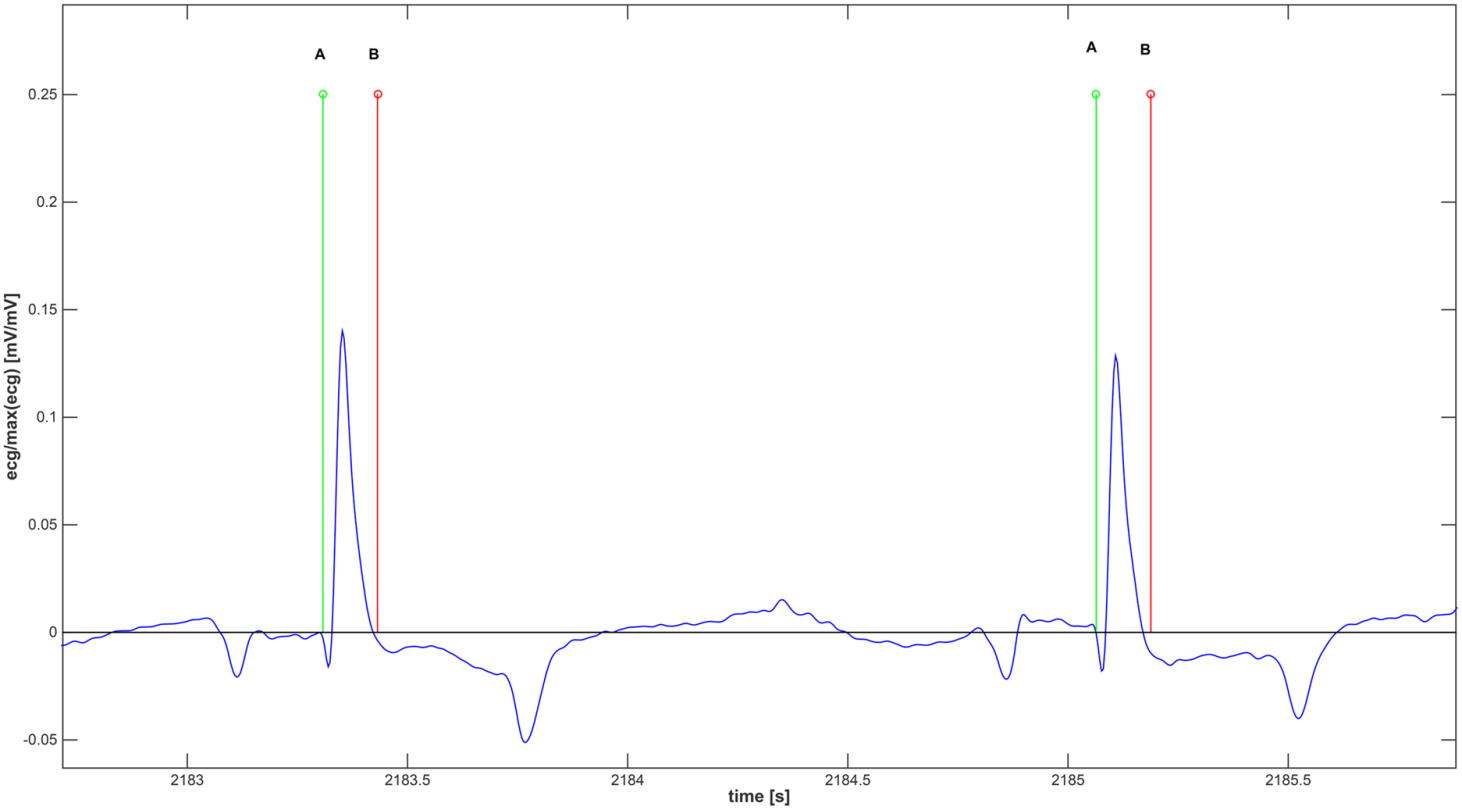
Example of two consecutive QRS complexes detected by the proposed method. Letter *A* indicates the stating time-instant while *B* indicates the ending time-instant.

**QRS Complex Detection** (QRSCD) used in this study is based on the computation of the energy of the second derivative of the ECG (see diagram reported in Fig 3). Operatively, the second derivative of the ECG signal was segmented by applying a 30 ms long moving window with a time shift equal to 15 ms, therefore with an overlap of 50%. Then, frame by frame, the energy signal was obtained as the sum of squares of the data within each frame. Afterwards, in each of non-overlapped time intervals lasting 1.5 s, Local Maxima (LM) and Local minima (Lm) of the energy signal were detected. By moving the window, a series of LM and Lm is constructed and used to estimate the threshold used to identify the QRS complexes. More specifically, after computing the medians of both the LM and Lm series, the threshold was estimated as the median value of the two previously calculated values. Of note, for an accurate computation of the LM and Lm, it is important that at least one ECG be present into the window, therefore we have chosen the value of 1.5 s, as it coincides with the maximum of the mean RR interval computed over 60 minutes among all of the 7 horses. Afterwards, energy segments of the derived signal showing a energy value greater than the threshold were considered as belonging to a QRS complex (see Fig 4). Fig 4 shows an example of QRS detection algorithm applied to two consecutive QRSs. Specifically, for each QRS, two markers (A and B, A starting time-instant and B ending time-instant of QRS) are computed. In order to test the performance of the QRS detection algorithm proposed here, 19 ECG records (Fs = 250Hz) gathered from the human QT database [61, 62] provided by PhysioNet [63] (PN-DB) were used. ECGs were chosen in order to collect a broad variety of QRS and ST-T morphologies. A total of 596 QRS complexes were detected and segmented by our algorithm and compared with the manual labeling provided by the database [64].

**Fig 5:**
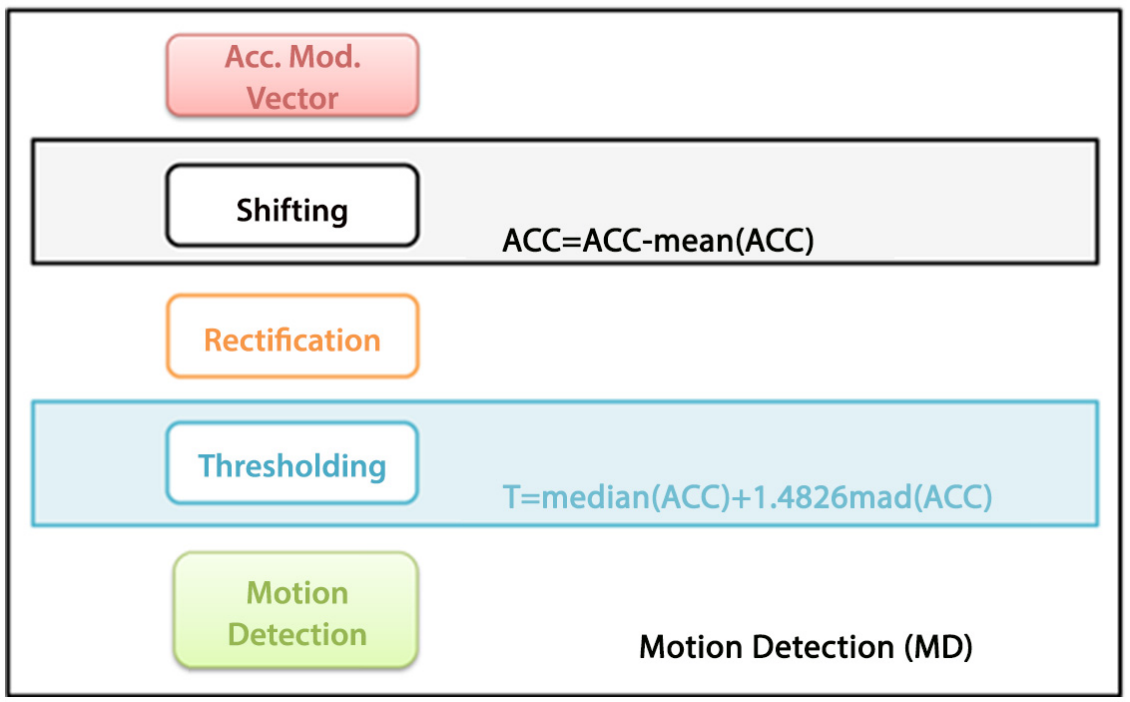
Block diagram of the Motion-detection procedure. After the signal is rectified and subtracted by its mean value, strong movement segments are detected through a threshold as estimated in Eq (2). These motion signal segments can introduce MAs.

**Fig 6:**
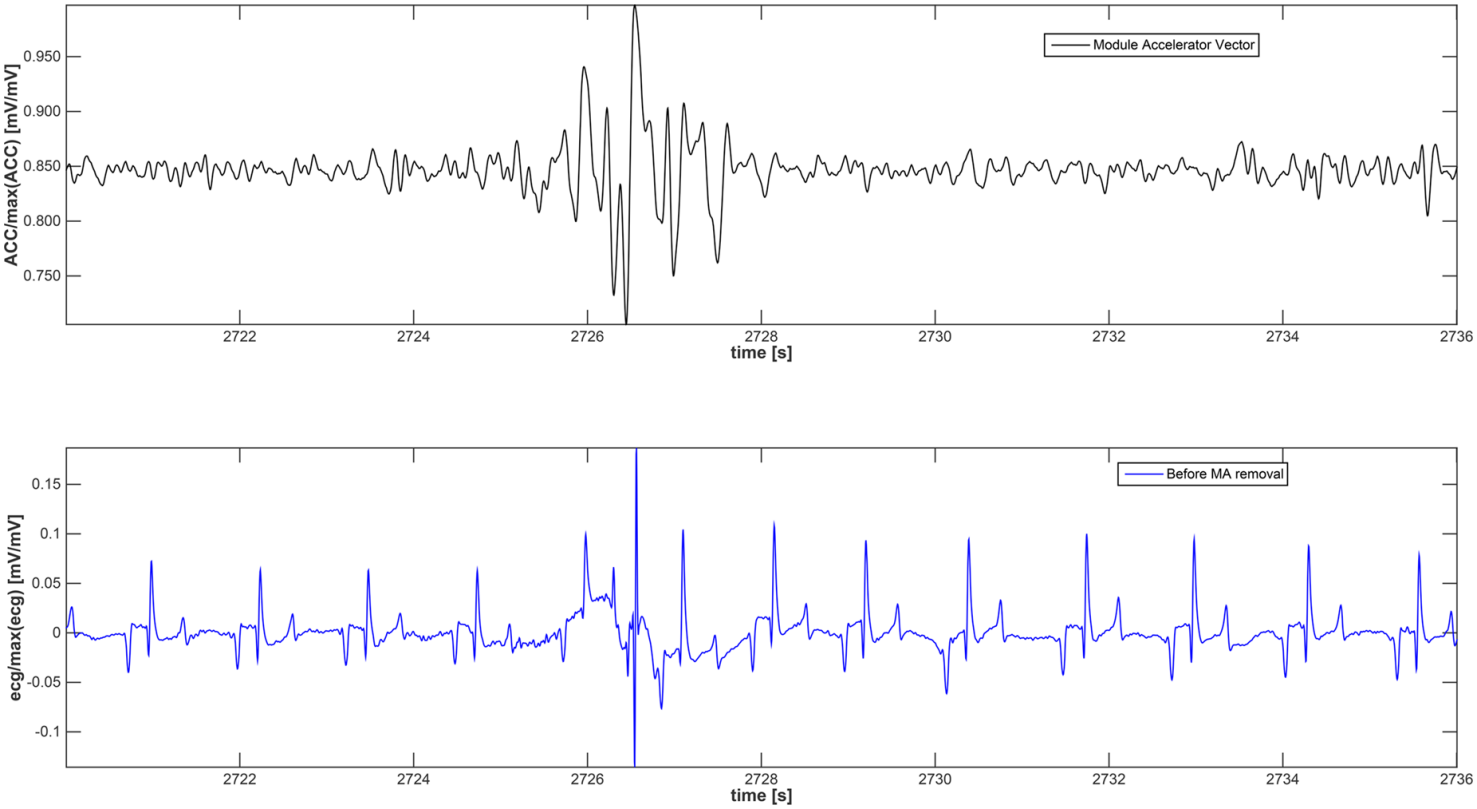
The graph at the top shows the acceleration module with an event of remarkable movement, while at the bottom the ECG with the corresponding MA is reported.

**Motion Detection** (MD) algorithm detects the time-intervals of ACC where the amplitude was supposed to generate MAs. Fig 5 shows a concise block diagram of the MD algorithm. The movements that can contribute to the MAs are computed by means of a threshold, *T*, that was estimated by considering the rectified acceleration according to:

$$\begin{array}{c} T=median(ACC)+1.4826*mad(ACC); \end{array}$$ (2)

where *median*(*ACC*) is considered as the robust estimate of the average of acceleration module, and 1.4826 ∗ *mad*(*ACC*) is used as robust estimate of the standard deviation of the acceleration module, where robustness is estimated according to [65]. The acceleration signal segments having a value lower than *T* Eq (2) are considered unable to generate MA in the corresponding ECG signal. Fig 6 shows the module of the acceleration vector (upper plot) and the ECG signal (lower plot) in the same time frame. Of note, this figure shows that information provided by the accelerometer, when a relevant movement occurs, is different from the ECG both in terms of shape and frequency contribution.

**Stationary Wavelet Reduction** (SWR) is based on the application of Stationary Wavelet Transform (SWT) to decompose ECG into different scales for separating small features (fine scales) from large features (coarse scales), [66]. Since SWT is able to perform local signal processing analysis, i.e., to zoom in on any interval of time or space, it could reveal hidden aspects of the data that other signal analysis techniques fail to detect. For implementing this decomposition it is essential to perform a redundant representation of the signal through an orthonormal transform to guarantee a time-invariance transform (i.e., the coefficients of a delayed signal are a time-shifted version of those of the original signal). For a given signal *x*(*t*) ∈ *L* ^2^(ℝ), the SWT can be obtained by integrating the product of the given function with the complex conjugate of the wavelet function *ψ*_*j*,*k*_(*x*)

$$\begin{array}{ccc} W_{j}^{k}(x) & ≜ & \frac{1}{2^{\frac{j}{2}}}\int_{-\infty }^{\infty }x\left(t\right)\psi_{j,k}^{*}\left(\frac{t}{2^{j}}-k\right)dt\\ & ≜ & \langle x\left(t\right),\frac{1}{2^{\frac{j}{2}}}\psi_{j,k}(\frac{t}{2^{j}}-k)\rangle \;\left(j,k\right)\in Z \end{array}$$ (3)

where *ψ*(.) is referred to as a mother wavelet, * stands for complex conjugation, and ⟨˙⟩ represents the inner product of the two functions, hence, the orthonormal wavelet basis *L* ^2^(ℝ) was $\left\{2^{-\frac{j}{2}}\psi \left(\frac{t}{2^{j}}-k\right),\left(j,k\right)\in \mathbb{Z}\right\}$. Then by performing the multiresolution analysis (MRA) as proposed by Mallat et al. [67], the signal was decomposed into various resolution levels. At each level the signal is decomposed into two sub-signals: the first, called *approximation*, contains information at lower frequencies, and the second, called *detail*, contains information at higher frequencies. Finally, detail coefficients *D*_*j*,*k*_ and approximation coefficients *A*_*j*,*k*_ at level *j* are given by

$$\begin{array}{c} D_{j}^{k}(x)\;≜\;\langle x\left(t\right),\frac{1}{2^{j/2}}\psi (\frac{t}{2^{j}}-k)\rangle \;\left(j,k\right)\in Z \end{array}$$ (4)

$$\begin{array}{c} A_{j}^{k}(x)\;≜\;\langle x\left(t\right),\frac{1}{2^{j/2}}\phi (\frac{t}{2^{j}}-k)\rangle \;\left(j,k\right)\in Z \end{array}$$ (5)

where *ϕ*(˙) is the scaling function. The mother wavelet and the scaling function must satisfy the two-scale equations:

$$\begin{array}{c} 2^{-1/2}\phi \left(\frac{t}{2}-k\right)=\sum_{n=-\infty }^{+\infty }h_{n-2k}\phi \left(t-n\right) \end{array}$$ (6)

$$\begin{array}{c} 2^{-1/2}\psi \left(\frac{t}{2}-k\right)=\sum_{n=-\infty }^{+\infty }g_{n-2k}\phi \left(t-n\right) \end{array}$$ (7)

where {*h*_*k*_}_*k*∈ℤ_ and {*g*_*k*_}_*k*∈ℤ_ are the impulse responses of lowpass and highpass paraunitary quadrature mirror filters (QMF’s), respectively [68]. Let define *ϕ*_*j*,*k*_(*x*) and *ψ*_*j*,*k*_(*x*) as follows

$$\begin{array}{c} \phi_{j,k}\left(t\right)=2^{-j/2}\phi \left(2^{-j}t-k\right) \end{array}$$ (8)

$$\begin{array}{c} \psi_{j,k}\left(t\right)=2^{-j/2}\psi \left(2^{-j}t-k\right) \end{array}$$ (9)

then $D_{j}^{k}\left(x\right)$ and $A_{j}^{k}\left(x\right)$ can be rewritten as

$$\begin{array}{c} D_{j}^{k}(x)=\left\langle x\left(t\right),\phi_{j,k}\left(t\right)\right\rangle \end{array}$$ (10)

$$\begin{array}{c} A_{j}^{k}(x)=\left\langle x\left(t\right),\psi_{j,k}\left(t\right)\right\rangle \end{array}$$ (11)

Therefore $D_{j+1}^{k}\left(x\right)$ and $A_{j+1}^{k}\left(x\right)$ can be obtained from Eqs (6), (7), (8) and (9) as

$$\begin{array}{c} D_{j+1}^{k}(x)=\sum_{n=-\infty }^{+\infty }h\left(n-2k\right)A_{j}^{k}(x) \end{array}$$ (12)

$$\begin{array}{c} A_{j+1}^{k}(x)=\sum_{n=-\infty }^{+\infty }g\left(n-2k\right)A_{j}^{k}(x) \end{array}$$ (13)

By doubling the number of input samples in the MRA, a redundant decomposition can be performed on the signal. Then the approximation and detail coefficients of SWT can be given by

$$\begin{array}{c} {\tilde{A}}_{j+1}^{k}(x)=\sum_{l=-\infty }^{+\infty }g\left(l\right){\tilde{A}}_{j}^{k+2^{j}l}(x) \end{array}$$ (14)

$$\begin{array}{c} {\tilde{D}}_{j+1}^{k}(x)=\sum_{l=-\infty }^{+\infty }h\left(l\right){\tilde{A}}_{j}^{k+2^{j}l}(x) \end{array}$$ (15)

where *h*{*l*} and *g*{*l*} are low- and high-pass defined orthogonal wavelet functions.

The idea behind SWT is the recursive application of a series of high- and low-pass filters to the data to produce two sequences: *approximation* and *detail*. In the theoretical fashion, the original signal can be decomposed into a set of infinite levels, but from a technical point of view, this number of coefficients must be limited (i.e., *M* = 2^−*j*^), where j corresponds to the order of the SWT. Different mother wavelets can be chosen to perform SWT analysis without changing the obtained results. Here, the Haar wavelet Eq (16) was chosen because it has the advantage of being numerically faster, theoretically simpler, physically easier to interpret and exactly reversible without any edge effects:

$$\begin{array}{c} \psi \left(t\right)=\{\begin{array}{cc} 1, & 0\leq t<1/2\\ -1, & 1/2\leq t<1\\ 0, & otherwise \end{array} \end{array}$$ (16)

In this work, a decomposition level equal to 5, as suggested by Li and Lin [69], was used to provide a good trade-off between the amount of available information and computational effort. The frequency content of obtained coefficient sequences strongly depends on the corresponding level. More specifically, low levels represent the fast changing parts of the signal, i.e. the QRS complexes, while high levels contain information about slower parts such as P and T waves.

#### Artifact Removal Phase

**Fig 7:**
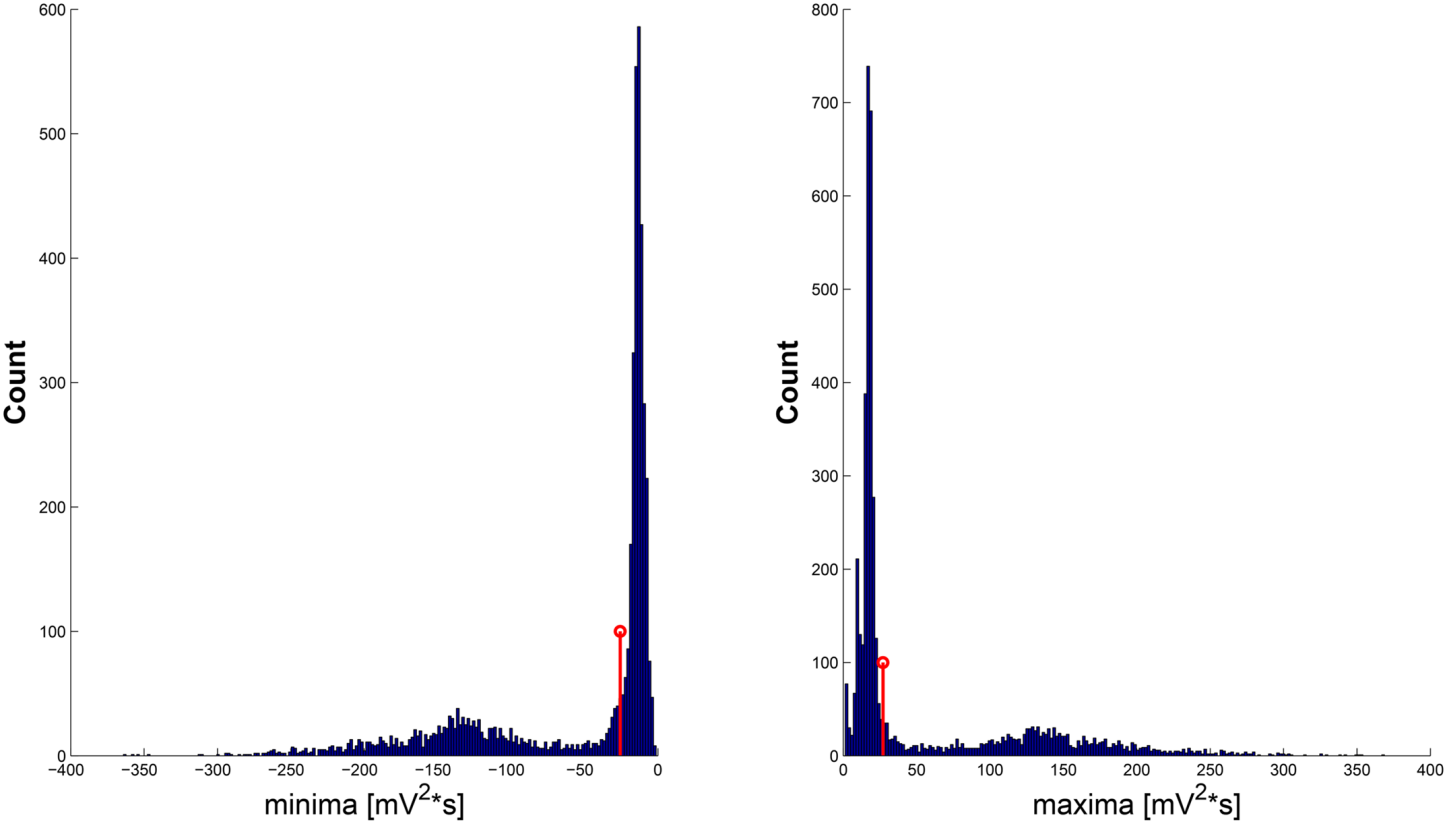
Histograms of the distribution of maxima (right) and minima (left) for a given decomposition level. In red the relative thresholds are displayed.

The Artifact Removal Phase (ARP) is mainly focused on the estimation of the wavelet components of the signal where MAs are mainly present. In particular, the algorithm computes two thresholds (Eqs (19) and (20)) for each decomposition level in order to recognize the signal affected by artifacts. By using the Motion Detection (MD) algorithm, we identified the time intervals in which strong movement contributions are present. ECG signal segments that were outside of these time intervals were considered artifact-free. Hence, we put intervals of the coefficients corresponding to the artifact-free ECG segments to zero, within the first three SWT decomposition levels, which are supposed to contain most of the informative contribution of the cardiac information. Afterwards, in all of the decomposition levels, we estimated maxima and minima of the wavelet coefficients within non overlapped frames lasting 1 second. Then, for these two distributions (i.e., maximum and minimum series) we robustly computed mean and standard deviation [65]. According to the statistics, the robust estimator of mean and standard deviation are:

$$\begin{array}{c} \mu_{i}=median\left(x\right); \end{array}$$ (17)

$$\begin{array}{c} \sigma_{i}=1.4826*MAD\left(x\right); \end{array}$$ (18)

where *x* is the investigated distribution, and *i* can assume only 1 or 2 for maxima and minima, respectively. Finally, obtained thresholds are expressed by

$$\begin{array}{c} M_{j}={\mu_{1}}_{j}+{\sigma_{1}}_{j}; \end{array}$$ (19)

$$\begin{array}{c} m_{j}={\mu_{2}}_{j}-{\sigma_{2}}_{j}; \end{array}$$ (20)

where *j* represents the decomposition level. In Fig 7 the distribution of maxima and minima, and the relative thresholds are displayed for a given decomposition level. Coefficients higher than *M*_*j*_ or lower than *m*_*j*_ are considered related to the movement artifacts. In performing the Inverse Stationary Wavelet Transform (ISWT) it is therefore possible to obtain a signal in the time domain corresponding to and depending on the MAs. Finally the movement artifact removal is performed by subtracting the obtained signal of the MAs from the original.

### Normalized Least Mean Square Adaptive Filter method

**Fig 8:**
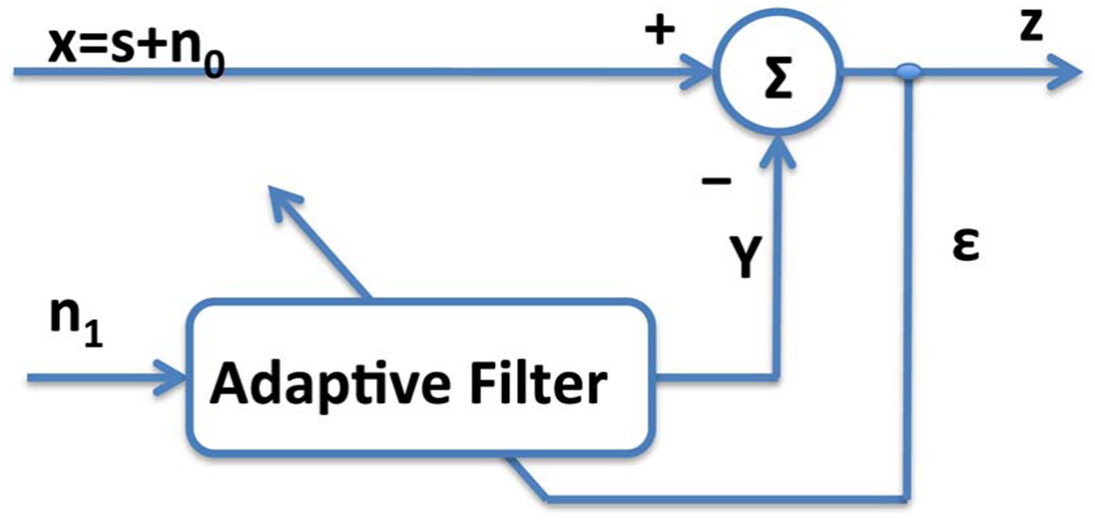
Recursive scheme of an Adaptive filter.

An adaptive filter has recursive structure where coefficients are continuously updated in the feedback loop, see Fig 8. The aim is to minimize the error, *e*(*n*), between the current filter output, *y*(*n*), and the primary input, *x*(*n*).

An adaptive filter, whose schema is reported in Fig 8, shows that there are two inputs, *x*(*n*) and *n*_1_(*n*), which are called the primary input and the reference input, respectively. The signal *x*(*n*) includes the desired signal *s*(*n*) plus undesired interference *n*_0_(*n*). Let us hypothesize that *s*, *n*_0_, *n*_1_ and *y* are statistically stationary and have zero means. Moreover, let us assume that *s* is uncorrelated with *n*_0_ and *n*_1_, and suppose that *n*_1_ is correlated with *n*_0_. The output *z* is obtained as follows:

$$\begin{array}{c} z=s+n_{0}-y \end{array}$$ (21)

By squaring, one obtains *z* ^2^ = *s* ^2^ + (*n*_0_ − *y*)^2^ + 2*s*(*n*_0_ − *y*) taking the expansion of both sides and considering that *s* is uncorrelated with *n*_0_ and *y*, we obtained:

$$\begin{array}{c} E\left[z^{2}\right]=E\left[s^{2}\right]+E\left[{\left(n_{0}-y\right)}^{2}\right]+2E\left[s\left(n_{0}-y\right)\right]=E\left[s^{2}\right]+E\left[{\left(n_{0}-y\right)}^{2}\right] \end{array}$$ (22)

The signal power *E*[*s* ^2^] will be unaffected as the filter is adjusted to minimize *E*[*z* ^2^]. Accordingly, the minimum output power is

$$\begin{array}{c} minE\left[z^{2}\right]=E\left[s^{2}\right]+minE\left[{\left(n_{0}-y\right)}^{2}\right] \end{array}$$ (23)

When the filter is adjusted so that *E*[*z* ^2^] is minimized, *E*[(*n*_0_ − *y*)^2^] is minimized as well. The filter output y is then a best least squares estimate of the primary noise *n*_0_. Moreover, when *E*[(*n*_0_ − *y*)^2^] is minimized, *E*[(*z* − *s*)^2^] is also minimized, therefore (*z* − *s*) = (*n*_0_ − *y*). As a consequence *z* is the best estimate of *s*.

### Statistical analysis

In this work several statistical tools were implemented to assess the performance of both the QRS complex detection algorithm and the SWMAR algorithm. More specifically, the first algorithm was applied to several ECG traces extracted from the PN-DB database with the aim of identifying the starting and ending time of each QRS complex. The outcome of the algorithm is, therefore, a series of time instants that was compared with that provided by the database itself. Comparison was performed by means of the Bland-Altman plot [70]. This plot is usually used to evaluate the agreement among two different measurements. In particular, we estimated both the mean difference, as estimated bias, and the Standard Deviation (SD) of the differences between the two series. The limits of agreement were computed as the average difference ±1.96 SD of the difference. If the differences were within mean ± 1.96 SD, the two methods could be considered interchangeably [71]. In addition, the regression line of the data was reported in the Bland-Altman plot to show any bias or trend. The evaluation of the performance of SWMAR algorithm in MA removal was performed through the statistical comparison of the outcomes of SWMAR, NLMSAF and the MA percentages of the raw signals before the treatment. In particular, statistics were performed through a nonparametric Friedman’s test applied to the three series of percentage to investigate any possible statistical difference among them. Moreover, a Wilcoxon signed rank test for paired samples was used to investigate any statistical difference among all of the couples of series. All the processing tools and statistical analysis were performed by means of *The* *MathWorks* − *Matlab*.

## Results

**Fig 9:**
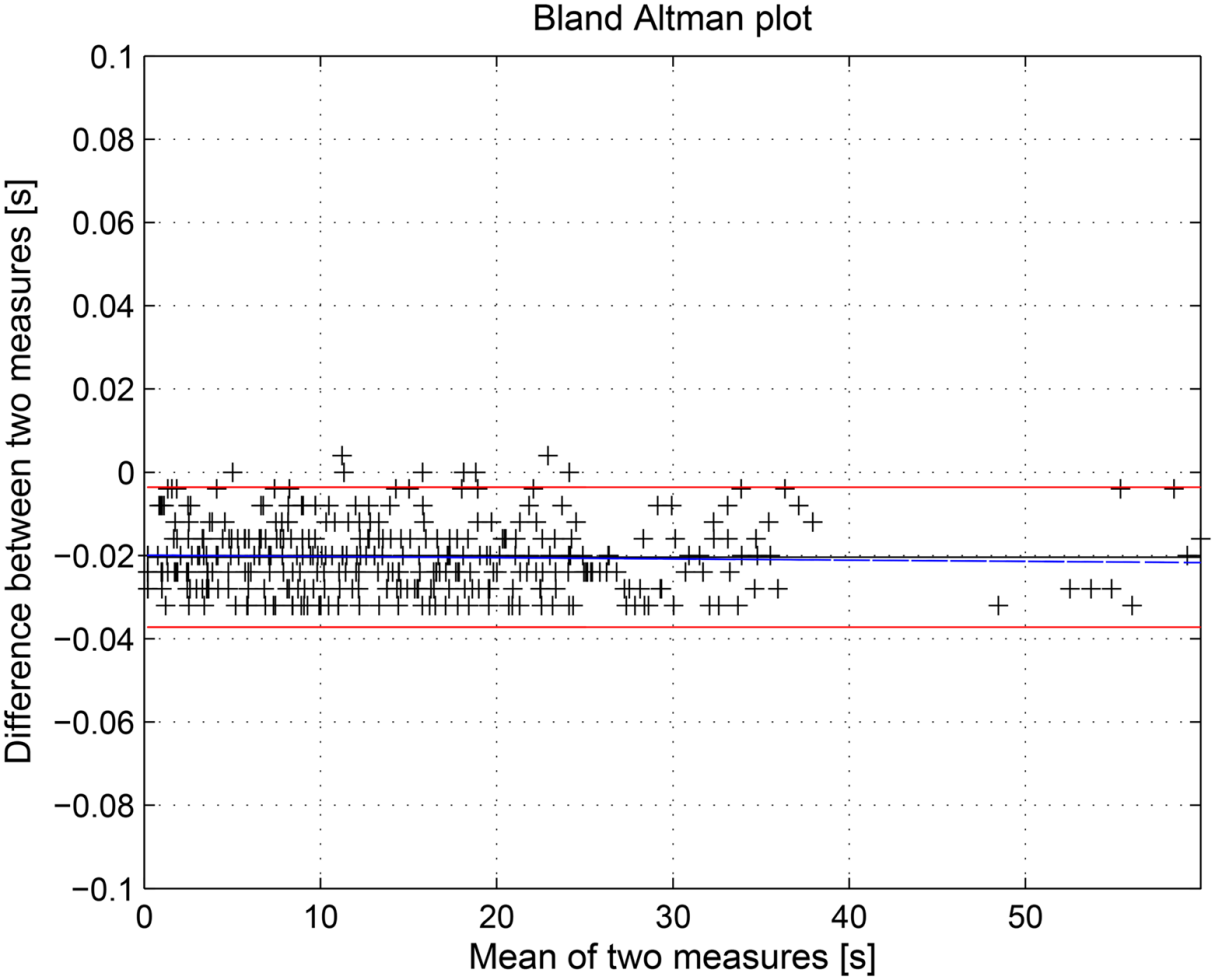
Bland-Altman plot comparing QRS complex starting time instant series between the SWMAR algorithm with those provided by PhysioNet Database. The black line indicates the bias (mean difference), the red lines are limits of agreement (mean ± 2 SD), whilst the blue line indicates the trend of the data. Mean = −0.0204 (95% CI: −0.0195 to −0.0213); limits of agreement between 0.0036 and −0.0372.

**Fig 10:**
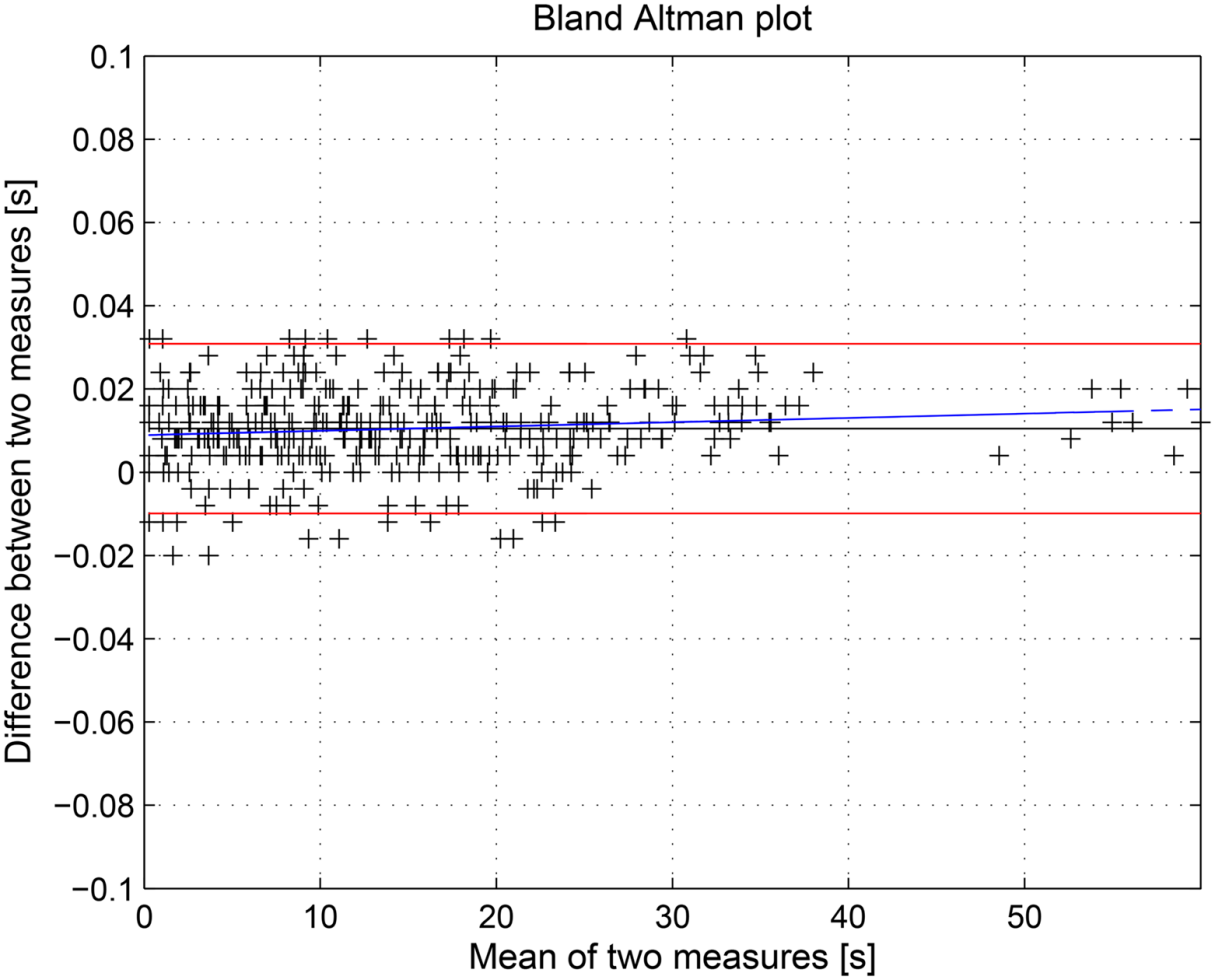
Bland-Altman plot comparing QRS complex ending time instant series between the SWMAR algorithm with those provided by PhysioNet Database. The black line indicates the bias (mean difference), the red lines are limits of agreement (mean ± 2 SD), whilst the blue line indicates the trend of the data. Mean = 0.0105 (95% CI: 0.0115 to 0.0094); limits of agreement between −0.0309 and −0.0099.

**Fig 11:**
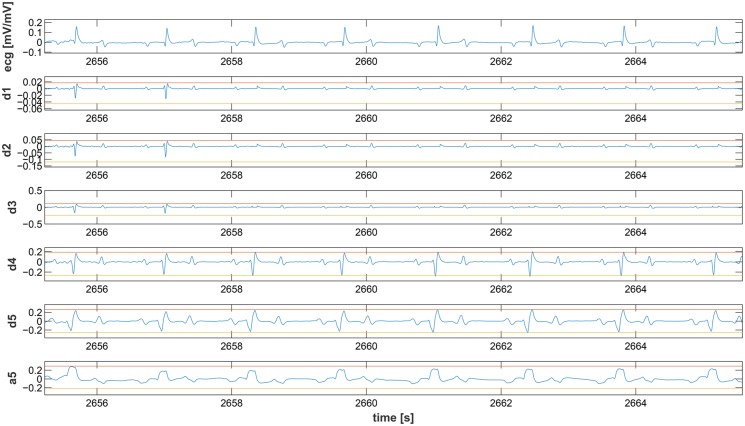
Decomposition of a clean ECG segment. At the top the original ECG signal is plotted, following the 5 decomposition detail signals and the approximation regarding the 5th level. Red and yellow lines represent the threshold levels as calculated according to Eqs (19) and (20).

**Fig 12:**
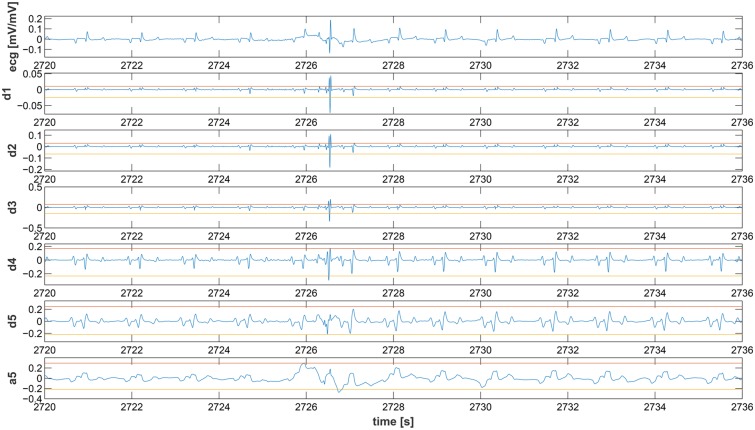
Decomposition of an ECG segment affected by artifacts. At the top the original ECG signal is plotted, following the 5 decomposition detail signals and the approximation regarding the 5th level. Red and yellow lines represent the threshold levels as calculated according to Eqs (19) and (20). The figure shows that MAs details and approximations coefficients that are out of the interval delimited by the two thresholds.

**Fig 13:**
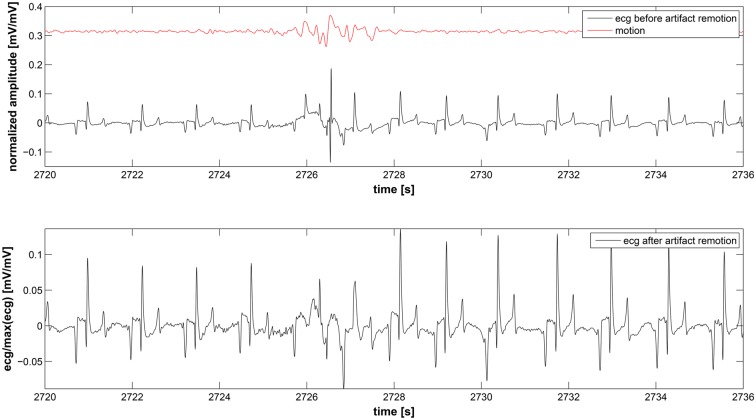
The upper plot reports the accelerometric signal (in red) along with the ECG signal with the corresponding artifact. The lower plot shows the ECG signal after applying the MA removal algorithm.

**Fig 14:**
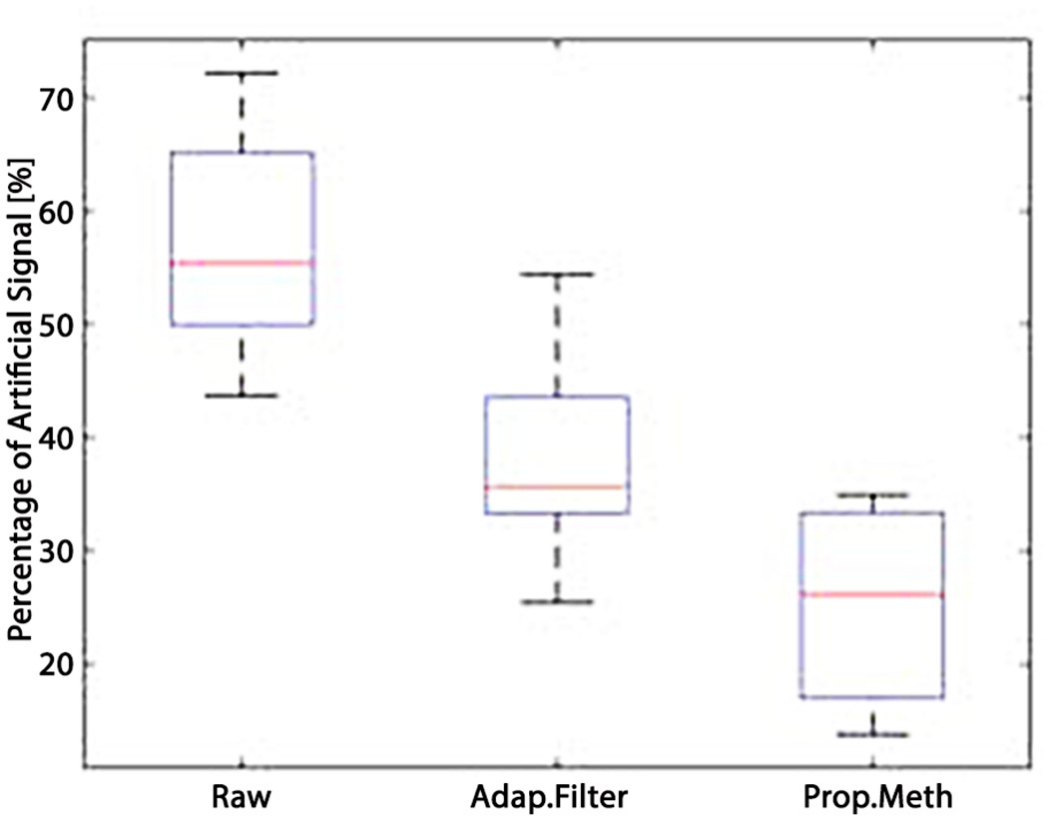
Boxplot of the MAs percentage before and after the method of removal MAs.

This section discusses the results obtained by applying the proposed methodology to seven hours of EGC acquired from horses. First, we evaluated the performance of the QRS detection algorithm by applying it to the online PN-DB. Afterwards, we investigated the performance of the SWT decomposition algorithm and, finally we evaluated SWMAR and NLMSFA applied to collected the ECGs comparing the results in terms of MA removal with respect to the starting MA amount present in the raw signals, where the MA were detected through visual inspection. More specifically, due to the novelty of the QRS detection algorithm, we preliminarily tested and validated it on the PN-DB. We applied the algorithm to ECG traces extracted from PN-DB and compared results. Figs 9 and 10 show the results of the Bland-Altman plots of the comparison results. More specifically, the mean of the differences between starting (ending) time-instant series calculated with the proposed algorithm and those provided by the PN-DB was −0.0204s (0.0105s) with SD 0.004. Moreover, Figs 9 and 10 show that most of the series points are within the limits of agreement for both comparisons. This means that the performance of our algorithm is comparable with those provided by the PN-DB [71, 72]. Concerning the performance of the SWT, Figs 11 and 12 show two examples of the decomposition of the ECG signals gathered from horses, with 5 decomposition levels. In Fig 11, a decomposition of ECG without MAs is presented whilst in Fig 12 a decomposition of an ECG affected by artifacts is reported. Comparing the two Figs, it is worthwhile noting that MA is much more prevalent at lower level of the decomposition, i.e. at higher frequency components. Fig 13 shows an example of the result of SWMAR applied to an ECG signal affected by artifacts. In the upper plot the motion signal (top) and the ECG with artifacts (bottom) are reported, while in the lower plot the ECG cleaned by the removal algorithm is shown. Moreover, the results of evaluation of the proposed method are shown in the Table 1. Specifically, it reports the percentages of detected MAs for the raw and the treated signals with SWMAR and NLMSFA. In addition, it also reports the difference in terms of MA percentage between raw signals and those treated with NLMSAF (*δ*_*N*_), and raw signals and those treated with SWMAR (*δ*_*O*_) in the seven hours of ECGs. More specifically, in the table the horses are labelled with the acronym *H* followed by a subscription, and the MAs are manually checked by two experts before and after the application of both methods. Results show that recognized MAs in all of the 7 horse-recordings after the application of the SWMAR and NLMSAF were reduced with respect to percentages of signal with artifacts obtained from RAW signals. In particular results obtained with the proposed method outperform those obtained by NLMSAF. Moreover, Fig 14 shows the box plot of detected MAs. A nonparametric Friedman’s test applied to the three series RAW, NLMSAF and SWMAR show a statistical significant difference with a *p*–*value* of 0.0021. Finally, Wilcoxon signed rank test for paired samples was used for comparing all the couples of classes. It reported significant differences in comparing raw data and both treated data, returning in both case a *p*–*value* equal to 0.0156. Moreover, a statistical significant difference was also reported in comparing the two treated data, showing an increased reduction of MAs (%) obtained with SWMAR (see Fig 14). In this latter case a *p*–*value* equal to 0.0313 was returned.

**Table 1:** Percentage (%) of detected MAs into: raw ECG signals (RAW); NLMSAF; the difference between raw and NLMSAF *δ*_*N*_; SWMAR; and the difference between raw and SWMAR.

Horse	RAW	NLMSAF	*δ*_*N*_	SWMAR	*δ*_*O*_
	(%)	(%)	(%)	(%)	(%)
*H*_1_	55.37	32.90	-22.47	15.00	-40.37
*H*_2_	55.31	34.53	-20.78	13.77	-41.54
*H*_3_	65.42	45.11	-20.31	34.91	-30.51
*H*_4_	43.69	35.64	-8.05	26.15	-17.54
*H*_5_	48.19	38.92	-9.27	22.93	-25.26
*H*_6_	64.10	54.36	-9.74	34.96	-29.14
*H*_7_	72.18	25.41	-46.77	28.69	-43.49

## Discussion and Conclusion

In several research studies the quality of ECG is considered empirically acceptable when distinct R-waves can be detected during the recordings. Detection can be facilitated by using automatic software, but more often than not it is done through manual and visual inspection. However, depending on heart rate and electrode position, the T wave of the equine ECG is commonly erroneously detected by commercial software as an R peak, thus manual inspection remains mandatory in this field [56]. Furthermore, manual inspection of the ECG trace could raise further issues, for example, the number of rhythm events could be dependent on observation periods, or intra- and inter-observer agreement for recognition and classification of arrhythmias during and immediately after exercise. Moreover, possible mistakes or disagreement could also be due to miscounting events in a large number of events [57]. In addition, computerised ECG interpretation programs have a high rate of misclassification, especially in non-sinus rhythm events [73]. The high number of physiological atrio-ventricular-blocks and high amplitude and variable morphology of the T wave make the use of such fully automated algorithms very difficult in horses [57]. The aforementioned considerations point out the importance and relevance of a specific algorithm for the reduction of MAs on the recording of equine electrophysiology of the heart. Moreover, since restriction of movement in horses dramatically increases their sympathetic activity, it is therefore recommended to be avoided [54]. This work underlines that quantity and quality of body movements is a fundamental variable that needs to be taken into account for the implementation of algorithms addressing the movement artifact issue [56].

A new algorithm was implemented in the acquisition of equine ECG signals in order to remove MAs. We integrated the time-invariance of the Stationary Wavelet Transform with the essential exogenous information of movement provided by a triaxial accelerometer. As a comparator, a NLMSAF was also applied to the same dataset. The outcomes of the two algorithms were manually evaluated by two experts and ranked as a percentage of MAs over the 7 hours of acquisition from horses (see Table 1). Algorithm performance was also evaluated in terms of reduction of the MA percentage. Referring to SWMAR, after the detection of the corrupted QRS complexes through the accelerometric data, the SWT was used in order to estimate the artifact-contribution to the ECG signals. After applying the proposed method to 7 hours of ECG recordings, results highlighted how the rates of artifacts detected by the physicians significantly decrease. In addition, as indicated by visual inspection, our algorithm achieved a good percentage of MAs removal with respect to NLMSAF. In fact, the boxplot reported in Fig 14 showed the statistics of the detected percentage of MAs on the raw data and after applying NLMSAF and SWMAR algorithms, respectively. The figure also shows that the SWMAR algorithm outperforms NLMSAF. As shown, the proposed method is reliable and effective, but it could fail if the ECG signal is heavily affected by movement artifacts whose amplitude is remarkable. Indeed in this case, the algorithm could be unable to exactly identify the starting and ending times of the QRS complexes. Next steps will be to test our algorithm on ECGs acquired from horses while they walk or trot outdoors. Nevertheless, the achieved results are promising in view of applying advanced processing techniques also to ECG equine signals otherwise not applicable due to the relevant presence of movement artifacts. It is worthwhile noting that the ECG has been already demonstrated to be nonlinear and non-stationary in humans, and has been studied in depth using both standard as well as nonlinear approaches. On the contrary, it has not been yet possible for horses, since long epochs of signal without movement artifacts cannot be acquired. In this view a system able to remove artifacts in equine-ECG would make it feasible to apply nonlinear approaches such as the Lyapunov exponent, entropy, or correlation dimension, providing important information for the biology of the equine cardiovascular system.

## Supporting Information

S1 Supporting Information Dataset. Electrocardiographic and Accellerometric data.
(ZIP)
