# Supplementary material for: A Novel Algorithm for Movement Artifact Removal in ECG Signals Acquired from Wearable Systems Applied to Horses
Source: PLoS One. 2015 Oct 20;10(10):e0140783. doi: 10.1371/journal.pone.0140783 (PMC4618928; doi:10.1371/journal.pone.0140783)
Supplement: S1 Supporting Information — Electrocardiographic and Accellerometric data. (ZIP) [file pone.0140783.s001.zip › Available Data/Legends and details of uploaded data.docx]

**Legend and details of uploaded data**

All of the electrocardiogram and accelerometers data spreadsheet files.

All data are presented as columns separated by a comma.

**File name**

The file name is constituted of name of the horse followed by a number representing the sensor used for the acquisition, as follows:

<Name of the horse>_<sensor>.txt

Here, it is an example of the uploaded files: “Elicona_AccX.txt”

The keyword, which refers to the sensor type could be expressed only by ‘1’ or ‘2’.

- ‘1’ represent the textile electrodes.

- ‘2’ represent the standard Ag/AgCl electrode

**File content**

Each file is constituted of two columns. The first column is the “timestamps”, which is the time instant at which the sensor value on the corresponding column refer to.

The second column is the value of the sensor. The value of the sensors can be either the accelerometer or the ECG value. Data are separated by a comma.

<timestamps>, <sensor-value>

Here , it is an example of the file content:

…..

51867.012, -460

51867.052, -441

51867.092, -426

51867.132, -435

51867.172, -428

51867.212, -435

51867.252, -433

51867.292, -434

51867.332, -437

51867.372, -444

51867.412, -430

51867.452, -437

51867.492, -450

51867.532, -447

51867.572, -451

51867.612, -410

51867.652, -492

51867.692, -536

51867.732, 5

……

……
